# COVID-19 Vaccine-Induced Thrombotic Thrombocytopenia: A Case Series

**DOI:** 10.7759/cureus.17862

**Published:** 2021-09-10

**Authors:** Adel A Alalwan, Aissar Abou Trabeh, Divya Premchandran, Mohamed Razeem

**Affiliations:** 1 Renal Medicine, Portsmouth Hospitals University NHS Trust, Portsmouth, GBR

**Keywords:** covid-19 vaccine complication, ad26.cov2.s vaccine, chadox1 cov-19 vaccine, vaccine-induced thrombotic thrombocytopenia (vitt), corona virus disease 2019, covid-19

## Abstract

Vaccine-induced thrombotic thrombocytopenia is a life-threatening prothrombotic syndrome that has been associated with two adenoviral vector-based coronavirus disease 2019 (COVID-19) vaccines. Although it remains a rare disorder with relatively low incidence, awareness of this condition is crucial given the ongoing vaccination programs of millions around the world. In this case series, we report four cases of vaccine-induced thrombotic thrombocytopenia, diagnosed at Queen Alexandra Hospital, Portsmouth, United Kingdom. We also review the mechanism of this syndrome, its clinical presentation, diagnosis, and course of treatment with emphasis on the role of therapeutic plasma exchange.

## Introduction

The coronavirus disease 2019 (COVID-19) catastrophe has led to over 212 million cases worldwide, and over four million deaths by August 2021 [1]. In the United Kingdom alone, over six million cases have been confirmed up to this point, and COVID-19 infection was listed as a culprit in around 155,000 deaths [2]. Vaccines are believed to be the most promising countermeasure for combating the COVID-19 pandemic and are being strenuously pursued. By late 2020, several vaccines had become available for use globally, over 40 candidate vaccines were in human trials, and at least 150 were in preclinical trials [3,4]. In the United Kingdom, 76.9% of the targeted population has received their second dose of the COVID-19 vaccine so far, which corresponds to almost 42 million individuals [2].

In February 2021, a prothrombotic syndrome was identified in a few individuals following COVID-19 vaccination, particularly with the ChAdOx1 CoV-19 vaccine (AstraZeneca, University of Oxford, and Serum Institute of India), and the Ad26.COV2.S vaccine (Janssen; Johnson & Johnson). This syndrome has been designated vaccine-induced immune thrombotic thrombocytopenia (VITT) [4-6]. The actual incidence of VITT remains largely unknown but seems to be exceedingly rare. Nevertheless, the large-scale vaccination programs of millions resulted in hundreds of cases being reported around the world, which have been associated with significant morbidity and mortality. Thus, awareness of VITT, its presenting characteristics, evaluation, and management, is essential given the challenging nature of these cases and the rapidly evolving therapeutic approaches.

## Case presentation

We report four individuals with VITT, who had positive platelet factor 4 (PF4) antibodies via enzyme-linked immunosorbent assays (ELISAs) after receiving their first dose of ChAdOx1 CoV-19 vaccine. A positive reaction for PF4 ELISA was established with an optical density of more than 0.4. The following laboratory parameters (reference range) were listed for each patient: the platelet count (150-410 ×10^9^/L), the activated partial thromboplastin time ratio (APTR) (0.8-1.2), the international normalized ratio (INR) (0.8-1.2), the fibrinogen level (1.5-3.5 g/L), and the D-dimer (0-0.5 ug fibrinogen equivalent units (FEU)/mL). All the patients had a negative polymerase chain reaction test for COVID-19 before hospital admission. They developed thrombosis with thrombocytopenia and were managed according to local and international guidance, in addition to an expert opinion of a hematologist [7,8]. Non-heparin anticoagulants, intravenous immunoglobin (IVIG), and oral glucocorticoids were administered to every patient. However, therapeutic plasma exchange was necessary in only two cases. 

Case 1

**Figure 1:**
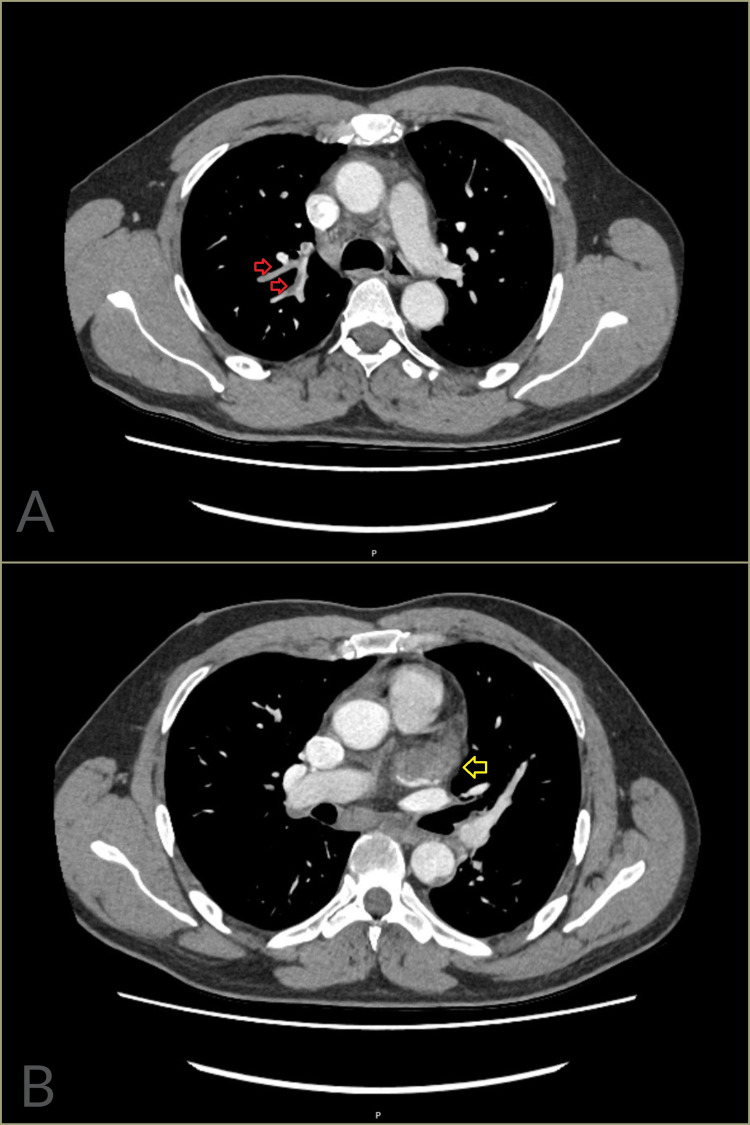
CT of the chest with contrast (Case 1). (A) There is reasonable opacification of the pulmonary arteries demonstrating thrombi in the right lobar branches (red arrows). (B) The left atrial appendage appears enlarged and is largely occluded by thrombus (yellow arrow).

A 47-year-old man, who has a history of hypertension and diverticular disease, presented seven days after vaccination with infero-posterior ST-segment elevation myocardial infarction requiring urgent percutaneous coronary intervention (PCI), thrombosis of distal right coronary artery (RCA) and the posterior descending artery (PDA), multiple pulmonary emboli, and left atrial appendage thrombus (Figures 1A, 1B). He underwent a PCI to the RCA; the PDA thrombus was conservatively managed. He did not receive heparin-containing products. He received tirofiban infusion post angiogram; however, this was stopped within two hours due to low platelet counts. On admission, the platelet count was 32 × 10^9^/L, the APTR was 1, the INR was 1.1, the fibrinogen level was 3.4 g/L, and the D-dimer level was 5.37 ug FEU/mL. The troponin level was remarkably elevated at 26,495 ng/L, with a reference range of 0-17.4 ng/L. The optical density of PF4 ELISA was 3.53. After expert hematology opinion, he was commenced on IVIG (1 g/kg) for two days followed by prednisolone 60 mg daily and anticoagulated with fondaparinux subcutaneous injection. One week after initial treatment, the platelet count remained below 50× 10^9^/L and the D-dimer level increased to 20.99 ug FEU/mL. Hence, he underwent five sessions of therapeutic plasma exchange with plasma (Octaplas). Argatroban infusion was used for anticoagulation during the exchanges. He remained on fondaparinux during this period. Following the last session of plasma exchange, the platelet counts normalized (160 × 10^9^/L), and the D-dimer level dropped to 4.11 ug FEU/mL. One week later, fondaparinux was converted to rivaroxaban. Prednisolone was tapered rapidly due to adverse effects including steroid-induced hyperglycemia. The patient was discharged on 10 mg prednisolone daily.

Case 2

**Figure 2:**
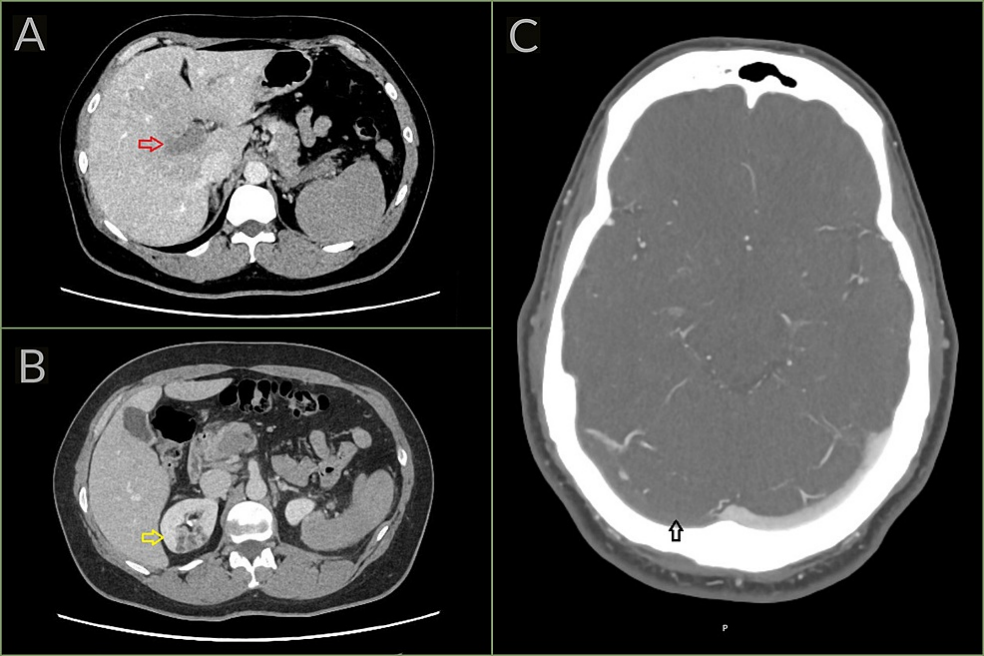
(A and B) CT of the abdomen with contrast and (C) CT of the head with venogram (Case 2). (A) There is complete occlusion of the portal vein and its branches (red arrow). (B) A wedge infarct is shown in the upper pole of the right kidney (yellow arrow). (C) The CT head with venogram (CTV) demonstrates occlusive thrombus within the right transverse sinus (black arrow). In comparison, the left transverse sinus is patent and completely opacified.

A previously healthy 48-year-old man presented five days after vaccination with the complete portal vein and splenic vein thrombosis, right kidney upper pole segmental infarct, acute right internal iliac artery thrombosis, and an occlusive thrombus within the straight sinus, inferior sagittal sinus, and right transverse sinus extending to the internal jugular vein (Figures 2A-2C). On admission, the platelet count was 18 × 10^9^/L, the APTR was 1, the INR was 1.2, the fibrinogen level was 2.3 g/L, and the D-dimer level was 66.6 ug FEU/mL. The optical density of PF4 ELISA was 2.05. He was immediately started on prednisolone 60 mg daily, given a single dose of IVIG (1 g/kg), and anticoagulated with fondaparinux. On the following day, therapeutic plasma exchange with plasma (Octaplas) was promptly arranged for five sessions; argatroban infusion was added during the exchanges. His fibrinogen level dropped after the second session of plasma exchange to 0.1 g/L and cryoprecipitate was given. Following the fifth session of plasma exchange, his platelet counts normalized (164 × 10^9^/L), and his D-dimer decreased to 16.94 ug FEU/mL. Fondaparinux was continued until he achieved therapeutic INR with warfarin. He was eventually discharged on warfarin and tapered dose regimen of prednisolone. 

Case 3

A 54-year-old man, with a previous history of Guillain-Barre syndrome two years ago, presented 15 days after vaccination with cerebral venous thrombosis (CVT) involving the left-sided transverse and sigmoid sinuses as well as the left jugular vein. The CVT was associated with recurrent tonic-clonic seizures for which he was kept on levetiracetam daily, requiring a short stay in the intensive care unit. On admission, the platelet count was 117 × 10^9^/L, the APTR was 1, the INR was 1.1, the fibrinogen level was 2.3 g/L, and the D-dimer level was 5.92 ug FEU/mL. The optical density of PF4 ELISA was 2.53. He was given IVIG (1 g/kg) for two days, commenced on prednisolone 70 mg daily for 10 days, and anticoagulated with fondaparinux. Six days later, he was discharged after being bridged to warfarin, and a tapered dose of prednisolone. On discharge, his platelet count was 195× 10^9^/L and the D-dimer level was 3.43 ug FEU/mL.

Case 4

A 29-year-old man, with a history of Janus kinase 2 (JAK-2) negative polycythemia, cardiomyopathy, and malabsorption, presented 12 days after vaccination with CVT affecting the transverse sinuses bilaterally and superior sagittal sinus. His CVT was complicated by an episode of tonic-clonic seizure and bilateral frontal venous infarcts. On admission, the hemoglobin level was elevated with a value of 189 g/L, the reference range being 130-170 g/L. The platelet count was 37 × 10^9^/L, the APTR was 1, the INR was 1.1, the fibrinogen level was 2.5 g/L, and the D-dimer level was 4.97 ug FEU/mL. The optical density of PF4 ELISA was 2.26. Following hematology consultation, he received a single dose of IVIG (1 g/kg), started on prednisolone 40 mg daily, and anticoagulated with fondaparinux. He also had a session of venesection on the sixth day of admission, due to persistent polycythemia. He was eventually discharged after two weeks in-hospital on warfarin and a tapered dose of prednisolone. On discharge, the platelet count was 198 × 10^9^/L.

## Discussion

COVID-19 vaccine-induced thrombotic thrombocytopenia (VITT) is a rare condition with a very low incidence rate. A small number of cases have been reported in the literature among the tens of millions of vaccinated individuals. Pavord et al. conducted a prospective cohort study involving patients with suspected VITT who presented to hospitals in the United Kingdom between March and June 2021. Among the 294 patients that were evaluated, 170 definite and 50 probable cases of VITT were identified. All the patients received the first dose of the ChAdOx1 CoV-19 vaccine and presented five to 48 days following vaccination [9]. In Norway, five cases had been reported from nearly 130,000 individuals vaccinated with ChAdOx1 CoV-19, suggesting an incidence of 1 in 26,000 [4,5]. In the United States, the centers for disease control (CDC) reported 15 cases from around eight million individuals vaccinated with Ad26.COV2.S, implying an incidence of 1 in 533,333 [6]. In addition, VITT has been reported to be life-threatening on numerous occasions with a mortality rate of approximately 22-60% [5,9-11]. In terms of risk factors, younger age (below 55 years of age) and females were proposed as possible risk factors based on initial reports [5]; however, more recent studies with a higher number of cases have shown no clear gender preponderance and no identifiable medical risk factors [9].

VITT is associated with high titers of immunoglobulin G class antibodies directed against platelet factor 4 (PF4; CXCL4). These antibodies potently activate platelets via platelet FcγIIa receptors [4,12]. Platelet activation in turn leads to the release of procoagulant microparticles and thrombosis. PF4 antibodies also mark platelets for removal by splenic macrophages which causes thrombocytopenia [10,12,13]. VITT closely mimics autoimmune heparin-induced thrombocytopenia both clinically and serologically, however, it's heparin independent. VITT is mainly associated with exposure to two adenoviral vector-based vaccines: ChAdOx1 CoV-19 and Ad26.COV2.S [4]. Greinacher et al. suggested that these adenovirus vector-based vaccines are at risk of inducing VITT through adenovirus and other PF4-DNA interactions. They also proposed that certain vaccine components such as EDTA and cell culture-derived human proteins, cause an acute inflammatory response that might be an important cofactor in VITT etiology [12].

The VITT prothrombotic syndrome starts in a narrow window five to 10 days post-vaccination in most individuals, leading to the identification of cases usually between five to 30 days following vaccination [4,10]. Thrombocytopenia may be an incidental finding or suspected based on the presence of petechiae or mucosal bleeding. Moreover, thrombosis has been the presenting feature in many of the reported cases of VITT, with both venous and arterial thromboses being described [5,6,9,10]. Thrombi are often present at multiple sites and in unusual locations. Venous thrombi can be found in the cerebral and splanchnic veins, as well as in the pulmonary arteries and deep veins of the legs. Arterial thrombi, albeit less frequent, can result in ischemic stroke, acute limb ischemia, and even myocardial infarction as in Case 1. The most common presentation reported in the literature is cerebral venous thrombosis, as noted in three of our patients [6]. The presenting symptoms usually reflect the site of thrombosis, for instance, Case 1 presented with chest pain and shortness of breath, Case 2 presented with acute abdominal pain and persistent headache. Cases 3 and 4 had headaches and seizures.

Consideration of both the clinical and the laboratory features is required for the diagnosis of VITT. A laboratory workup including a complete blood count with platelet count, coagulation profile, D-dimer, and fibrinogen levels is necessary. VITT is probable with platelet counts below 150 × 10^9^/L, D-dimer levels more than 4 ug FEU/mL, or low to low-normal fibrinogen levels [8]. Positive PF4 antibodies on ELISA testing are confirmatory [7,8]. However, appropriate treatment including the administration of non-heparin anticoagulant, and IVIG treatment should not be delayed while awaiting the results of confirmatory testing, particularly in individuals with high suspicion of VITT [4].

The management of VITT is an area of rapid evolution, and despite having published international and local guidelines, input from the consulting hematologist or other hemostasis and thrombosis expert is essential to help with the evaluation and management of these cases. Therapeutic anticoagulation is one of the primary treatments for VITT and is used unless there is a contraindication such as major bleeding. Anticoagulation with a non-heparin anticoagulant such as fondaparinux, argatroban, or a direct oral anticoagulant (DOACs) (e.g., apixaban, rivaroxaban) is advised. Argatroban is preferred during plasma exchanges because it's short-acting, administered by continuous infusion, and can be monitored and adjusted by checking APTR levels [4,7,8]. Anticoagulation can be continued for three months following normalization of the platelet count in cases of VITT with thrombosis, as long as no further thrombosis occurs [4]. High-dose IVIG (e.g., 0.5 to 1 g/kg intravenously daily for two days) has also been shown to be effective in the treatment of VITT along with anticoagulation. IVIG can interrupts VITT antibody-induced platelet activation [7,8,14]. In addition, glucocorticoids (e.g., prednisone 1 to 2 mg/kg) can be initiated, especially in cases with a platelet count of less than 50 × 10^9^/L. It’s also advised to avoid platelet transfusions unless necessary, such as in patients where urgent surgery is required [7,8].

Therapeutic plasma exchange has been proposed as rescue therapy in cases with refractory VITT that are unresponsive to standard therapies. It allows for the removal of anti-PF4 antibodies and immune complexes from the patient’s plasma, and for the replacement of factors consumed during the process of thrombosis. It also depletes factors supporting inflammation, including excess inflammatory cytokines in the circulation, as well as the extracellular vesicles which are primarily derived from activated platelets and support thrombosis [15,16]. Therapeutic plasma exchange should be continued until platelet normalization in refractory cases, and the recommended replacement fluid is 1:1 with plasma from healthy donors [11,15]. In Canada, therapeutic plasma exchange using full plasma (or half plasma and half albumin) as the replacement fluid resulted in halting the thrombotic process in a series of three patients with VITT who had persistent thrombocytopenia and extensive thrombosis despite treatment with anticoagulation and IVIG. Plasma exchange was performed daily for five to seven days, and all patients had a marked reduction in D-dimer levels and improvement in platelet counts following the exchanges. In one case, IVIG was added after therapeutic plasma exchanges 4 through 7, and in another case, one dose of rituximab was given after the fifth plasma exchange [11]. In the UK, Pavord et al. reported that 17 (8%) out of 220 patients with definite or probable VITT were treated with plasma exchange. They noted that therapeutic plasma exchange was associated with a survival rate of 90% in patients with severe thrombocytopenia and cerebral venous thrombosis, or severe thrombocytopenia and extensive thrombosis [4,9]. In our case series, Cases 1 and 2 showed dramatic improvement after completing five sessions of plasma exchange. In both patients, the platelet counts normalized (> 150 × 10^9^/L), and the D-dimer levels decreased by at least 70%.

## Conclusions

COVID-19 vaccine-induced thrombotic thrombocytopenia (VITT) is a rare but serious medical condition that should be promptly recognized for appropriate evaluation and management. It should be suspected in any individual who develops symptoms of thrombosis or thrombocytopenia during an appropriate time frame following one of the implicated vaccines.

The management of VITT is frequently complex and challenging, and an expert opinion is often needed. Our observations support an initial treatment with non-heparin anticoagulant, intravenous immunoglobins, and glucocorticoids if no immediate contraindications are found. Therapeutic plasma exchange can be reserved for refractory cases, and for cases with severe thrombocytopenia and extensive thrombosis.
